# On the Influence of Climate upon the Human Teeth

**Published:** 1857-01

**Authors:** 


### ARTICLE VII.

### *On the Influence of Climate upon the Human Teeth.*

So far as our existing knowledge extends, we have no satisfactory evidence that climate *per se* exercises a marked degree of influence over the strength or durability of the teeth. Among all savage or nomadic tribes these organs are proverbially strong and healthy, although hourly subjected to tests from which their more cautious civilized possessors would absolutely recoil. But we have undoubted evidence that the teeth of the latter are indirectly affected by change of climate and residence. Whether produced through sympathy with the body generally, by local action, or forms of dieting unadapted to the nature of the climes in which they may chance to dwell, are subjects well worthy the most careful and practical consideration. On the present occasion, however, we shall confine ourselves to the most common kind of injury, which appears to result purely from errors of diet, or the taking of food either in excess or in opposition to the rules of climate and temperature.

There is an excellent distinction drawn, in John Hunter's still valuable "Treatise on the Natural History and Diseases of the Human Teeth," between *mortification* and *simple death* of a part; and it is to the latter that our present brief observations will refer. It is hoped that this attempt to grapple with so difficult a subject as that of climateric influence, may lead others to investigate it also, in order that we may arrive at some practical solution of a question of such vital and enduring importance to society.

If we take the two largest receptacles of the expatriated Anglo-Saxon race—America and Australia—solely by way of example, we find that one of the most marked signs of physical degeneracy, if it really be such,* is in the early decay of the teeth. In this respect both continents suffer alike, whilst the aboriginal tribes of each are remarkable for the opposite tendency. Whether the latter would suffer in much the same way by residence in our climate, we have as yet had little means of ascertaining; but it is probable that, were the savage to forsake his *varied* character of food, and confine himself to animal diet here, the same result that accrues to us in his climate would happen to him. Yet this early destruction of the teeth can hardly be said to be peculiar to the first generation of white men, but rather to their immediate descendants. The saddest aspect of this affection is presented in the very early age at which the teeth begin to disappear. In our own experience of the above and several other tropical and extra-tropical portions of the globe, we found that the European, who engrafted himself there, did not suffer from this decay, but, on the contrary, in many instances preserved these valuable members in calmness and strength, so to speak, and with a freedom from many severe neuralgic symptoms which had long distressed him in the land of his birth. It was not so with his descendants. Several examples were brought under our notice where "stumps" that had long harrassed their possessor in a colder latitude had remained quiescent in a more genial clime, even up to the latest period

* See Dr. Knox's "Races of Men," for the hypothesis that the white man's descendants would degenerate and disappear entirely from North America were it not for the infusion of new blood by the large annual European immigration.

of existence. Yet in the latter cases *decay* had surely and slowly proceeded, but with hardly any trace of inflammatory action, save that which apparently accrued upon temporary exposure. There was, in truth, the *simple death* of the parts, described by Hunter and others.

What is generally understood as "tooth-ache" is a rare affection in the warmer latitudes; but in these, and especially such as are exposed to great and severe alternations of temperature, attacks of pain and swelling of the gums, particularly of the lower jaw and cheeks, are not uncommon, although they are found to subside with a rapidity very similar to those common attacks which supervene upon cold and exposure to wind and rain, setting in as they do with a turbulence peculiar to these places. In such cases, so long as the malady lasts, and that may not be more than ten or fifteen hours, the irritation of the whole mass of the gums may be extreme, and as this gradually subsides, a numbness or want of vital tone in the parts is experienced, the mucous surface of the mouth being spongy and cold, until a reaction takes place, saliva is secreted freely, and the parts regain their pristine health and vigor.

To attribute these circumstances of local action to symptoms either of ordinary neuralgia, acute rheumatism, or genuine dyspepsia, would hardly hold, inasmuch as the subjects of these attacks were not, nor had been, the victims of dyspepsia, nor of the other enumerated forms of complaint. The cause originated undoubtedly in climate, aided by errors of habit, dress, and living, which may be fairly charged against Europeans. The results of all this upon the future health of the teeth may be partly opined, but with our present slight knowledge of these phenomena can barely be explained. Did the after *simple death* of the teeth, either consecutively or altogether, that is, so far as to external view, they exhibited signs of positive decay, proceed from such attacks, or from the same causes that destroy these organs irrespective of locality or temperature? We think neither one nor the other, and for these reasons. The great error of most Europeans, and especially our own countrymen, who have migrated to hot climates, is the abuse, not so much of alcoholic drinks as of *animal* food—the latter often leading on to the former. So far as we could trace, there was scarcely one in a hundred who had sustained severe losses of teeth who had not eaten more animal food daily than the wants of his or her bodies, owing to the change of climate, demanded. The effect of this is more strikingly exemplified in residents of towns, whose lives are more indolently and luxuriously passed than are those of the people who reside in the country; and in the fair sex, especially, this serious disfigurement is sadly prominent.

Nothing can be more unlikely than the supposition that this loss of teeth proceeds from the scrofulous diathesis. Scrofula is not a disease of warm or tropical latitudes, but of temperate and colder climes, whilst the removal of the patient from the latter to the former is one of the surest methods of cure we possess. We may consider it, then, as a simple and gradual *death of the parts*, produced by some morbid condition of the mucous lining of the mouth and of the alimentary canal generally, resulting from the over-stimulating or fermenting action of proportionate excess of animal food. So long as this habit is persevered in, no art can stay the certain destruction of these organs, as if, in the providence displayed in nature, a source of aliment unsuited to the bodily requirements was only to be discarded after the destruction of those very members by which alone it could be prepared for the stomach! Surely the loss of one tooth by this silent process should be a timely warning to all European residents in hot countries; and if a corrective be possible on the part of the medical attendant upon such transgressions, occasional powerful laxatives, followed by cooling medicines, may, by removing the offending matter, stay an evil that more philosophic reasoning appeals against in vain. The aboriginal denizen of those lands purchases his meed of animal diet at the cost of immense exertion, with its attendant fatigue, and at certain seasons is confined entirely to the products of the vegetable world, seasoning his frame also with those long compulsory fasts which are neither known nor permitted by his more civilized brethren. The savage eats largely and voraciously, but the foregoing peculiarities of his mode of existence, temper this spirit, and there is no superfluity of dross or garbage, fermenting within a frame so active and impulsive as his own. It is not so with his fellow-man, bearing around him, whithersoever he may wend his way, all the blessings and adjuncts of civilization ; he, on the contrary, sets his own rules against the very laws of nature, and although he may not be "digging his own grave with his teeth," he is assuredly reversing that old and familiar saying by doing the same sepulchral office for those worthy and indispensable members of his body.—*Ib.*

